# VIEWS ON AGING ALTER THE IMPACT OF DEATH FEARS ON GENERALIZED ANXIETY

**DOI:** 10.1093/geroni/igz038.1706

**Published:** 2019-11-08

**Authors:** G Rainville, Laura Mehegan

**Affiliations:** AARP, Washington, District of Columbia, United States

## Abstract

This study looks at the relationship between fear of death, views on aging, and generalized anxiety. We theorize that positive views on aging (such as disagreeing that old age is a time of loss and decline) will buffer anxieties related to the unavoidable event of death. To test an implication of this theory, we ran a model looking at whether scores from a five-item views of aging scale altered the relationship between a fear of death measure (i.e., Collet-Lester’s “Your death” subscale) and generalized anxiety as measured on the GAD-7. Covariates related to religious beliefs, history with depression and anxiety, stress, social desirability reporting, and routine behaviors were included. An online probability sample of American adults age 18 and older (fielded January 2019) yielded results consistent with a “views-on-aging-as-an-anxiety-buffer” theory. Specifically, visualizations indicate that in a high fear of death condition, those with positive views of aging have significantly lower anxiety levels than those who do not. As such, this research suggests that positive views on aging may console those harboring fears about death.

